# Prognostic significance of programmed cell death 1 expression on CD8+T cells in various cancers: a systematic review and meta-analysis

**DOI:** 10.3389/fonc.2024.1531219

**Published:** 2025-01-14

**Authors:** Zhiyong Wan, Meng Cui, Jia Yang, Dan Liao, Junliang Chen, Fanmin Li, Yin Xiang, Zhiwei Cui, Yang Yang

**Affiliations:** ^1^ Department of General Practice, People’s Hospital of Leshan, Leshan, China; ^2^ Department of Medical Laboratory, People’s Hospital of Leshan, Leshan, China

**Keywords:** PD-1+CD8+ T cells, overall survival, progression-free survival, disease-free survival, cancer

## Abstract

**Background:**

Increased PD-1 expression on CD8+ T cells is considered as a hallmark for T-cell exhaustion, and is thought to be related to the prognosis of cancer patients. However, discrepant results have made it difficult to apply PD-1+CD8+T cells and tumor prognosis to clinical practice. Therefore, we conducted a meta-analysis to evaluate its prognostic value in human cancers.

**Methods:**

PRISMA reporting guidelines were strictly followed for conducting the current meta-analysis. The PubMed, Web of Science, Embase databases were searched from inception to November 2024. The pooled Hazard Ratio (HR) along with 95% confidence intervals (CIs) of each article were combined for the associations of PD-1+CD8+ T cells with overall survival (OS), progression- free survival (PFS) and disease-free survival(DFS). Subgroup analyses were performed for area, specimen type, cancer type, treatment, detected method and cancer stage.

**Results:**

A total of 20 studies (23 cohorts, 3086 cancer patients) were included in our study. The expression PD-1+CD8+ T cells in cancer patients tended to predict poor overall survival (OS) (HR: 1.379, 95%CI: 1.084-1.753, *p*= 0.009), and unfavorable disease-free survival(DFS) (HR: 1.468, 95%CI: 0.931-2.316, *p*=0.099), though it did not reach statistical significance. Begg’s and Egger’s test demonstrated that no obvious publication bias was exist.

**Conclusions:**

High PD-1 expression on CD8+ T cells is associated with worse survival outcomes, which can be potentially used as a prognostic marker of malignant tumor.

## 1 Introduction

Immune cells are known to be determinants of tumorigenesis, progression and metastasis, which play an important role in tumor elimination, surveillance and escape (1). Among these immune cells, cytotoxic CD8 + T cells represent the main anti-tumor TIL population and are considered as a positive prognostic factor in the majority of tumors. However, some studies found that the successful clearance of cancer cells by tumor-infiltrating lymphocytes (TILs) is impeded by a series of immune inhibitory mechanisms that are active in the tumor microenvironment (2–4), including the upregulation of immune checkpoint proteins such as PD-1, CTLA-4, Tim-3 and Lag-3 on TILs (5).

Programmed death-1 (PD-1) is a surface receptor expressed by lymphocytes that suppresses its proliferation and effector function by binding to PD-1 ligands such as B7-H1 (also known as PD-L1) and B7-DC (also known as PD-L2) expressed on other cells (6). Recently, some studies have showed T cells expressing these checkpoint molecules are considered to be characterized by a state of dysfunction, accompanied with loss the ability of cytokine production (IL-2, TNF-α and IFN-γ) and killing capacity, termed T cell exhaustion (7–9). A study pointed out that the tumor-infiltrating effector CD8+ T cells showed a drastic increase in PD-1 expression, and PD-1 upregulation promoted CD8+ T-cell apoptosis and postoperative recurrence in hepatocellular carcinoma patients (10). Although PD-1 can be expressed on any T cell during activation, it is frequently linked to the exhaustion of CD8 + T cells (11). Therefore, increasing researchers focused their attention on the role and activation status of infiltrating CD8+ T-cells including PD-1 expression in these immune cells.

Whether the expression of PD-1 on CD8+T cells can be used as a prognostic indicator has been explored. Hsu and his colleagues (12) found that increasing of programmed death-1-expressing intratumoral CD8+ T cells predicted a poor prognosis for nasopharyngeal carcinoma. In addition, according to Ma’s research, CD8 + T cells were classified intoPD1 Hi, PD1 Int and PD1-, PD1 Hi CD8 + T cells highly expressed exhaustion-related inhibitory receptors (TIM3, CTLA-4, etc.) and transcription factors (Eomes, BATF, etc.), and PD1 Hi CD8 + T cells were significantly correlated with poor prognosis (13). While some objections have been raised, they suggested that high proportion of PD-1 in CD8+ tumor-infiltrating T-cells improved survival outcomes in some cancers. Pokrývková *et* (14) indicated that increased expression of PD-1 in CD8+ T cells conferred improved survival outcomes, and PD1 + CD8 + Cells are an independent prognostic marker in patients with head and neck cancer. These results indicated a controversial prognostic value of PD-1+CD8+ T cells in human cancers.

The inconsistent results may be due to the different area, specimen type, cancer type, anti- cancer therapy, detected method and cancer stage. Hence, we conducted a meta-analysis to systematically assess the prognostic role of PD-1+CD8+ T cells in various cancers.

## 2 Materials and methods

### 2.1 Literature search and search strategy

The preferred reporting items of the Systematic Review and Meta-Analysis (PRISMA) Reporting Guidelines are strictly followed to document current meta-analyses. To verify compliance with established guidelines, the PRISMA checklist has been incorporated into **Supplementary Table S1** as a key tool for determining compliance. The protocol for the meta-analysis was registered at the International Platform of Registered Systematic Review and Meta-analysis Protocols (INPLASY) and assigned the registration number INPLASY2024110075. We systematically searched the PubMed, Web of Science, Embase databases prior to November 2024. The search key words were as follows: (((((((cancer[Title/Abstract]) OR (carcinoma[Title/Abstract])) OR (neoplasm[Title/Abstract])) OR (tumor[Title/Abstract])) OR (tumor[Title/Abstract])) AND ((pd-1[Title/Abstract]) OR (programmed cell death 1[Title/Abstract]))) AND (CD8+ T cell[Title/Abstract])) AND (prognosis[Title/Abstract]). Studies investigating the association between PD-1 expression on CD8+ T cells and cancer patient survival is currently a candidate for our meta-analysis. Besides, manual review was conducted on the references of relevant articles for additional candidate studies. The search procedures and related keyword combinations are documented in **Supplementary Table S2**.

The PICOS framework was as follows:

Population: This study involved patients who were diagnosed with cancer.

Intervention: The intervention under consideration was the presence of programmed cell death 1 expression on CD8+ T cells.

Comparison: The comparison was conducted to assess the presence of programmed cell death 1 expression on CD8+ T cells on survival.

Outcomes: OS and/or PFS and/or DFS in the presence of programmed cell death 1 expression on CD8+ T cells.

Study design: Case−control.

### 2.2 Study selection and inclusion-exclusion criteria

In order to search for relevant articles, two independent reviewers first screened the title and abstract of the article, and then conducted further access to the entire article. Differences among reviewers are resolved through discussion or negotiation with a third researcher until consensus is reached. Articles were included if they met the following inclusion criteria: (1) the researcher population must be diagnosed cancer patients; (2) at least one of these patient groups must detect PD-1 expression on CD8+T cells; (3) the study population should include the hazard ratio (HR) and 95% confidence interval (95% CI) of PD-1+CD8+T cells associated with OS and/or PFS and/or DFS, or provide sufficient data to calculate HR and 95% CI. Besides, the exclusion criteria were as follows: (1) reviews, letters or case reports; (2) animal or cell-line articles; (3) accompanied by other detection markers; (4) inefficient in providing data for calculate HR and 95% CI.

### 2.3 Data retrieval and quality assessment

Two researchers extracted information from eligible studies, and the information was as follows: first author, publication year, country, number of samples, specimen type, anti-cancer therapy, cancer stage, HR, and 95% CI of survival outcomes. In addition, to evaluate the quality of the included articles, the Newcastle–Ottawa scale (NOS), which was used to assess the quality of cohort studies and case–control studies. The highest score is 9 points, and studies with a score of six or more are considered to be high-quality (15). Quality assessment was presented in **Supplementary Table S3**.

### 2.4 Statistical analysis

Stata 12.0 was adopted for the meta-analysis, and p<0.05 was considered statistically significant. HR for OS, PFS and DFS and 95% CIs were pooled to measure the relationship between time and events. Heterogeneity was assessed by Cochran’s Q and I-squared (I^2^) tests. I^2^>50% with p value of Q test <0.1 indicated significant heterogeneity, and then a random- effect model was applied to combine HR and 95% CI of survival outcomes. Otherwise, a fixed-effect model was used. In addition, to find the source of significant heterogeneity, sensitivity analysis and subgroup analysis were performed. Begg’s and Egger’s test were used to assess publication bias.

## 3 Results

### 3.1 Literature retrieval and selection

**Figure 1:**
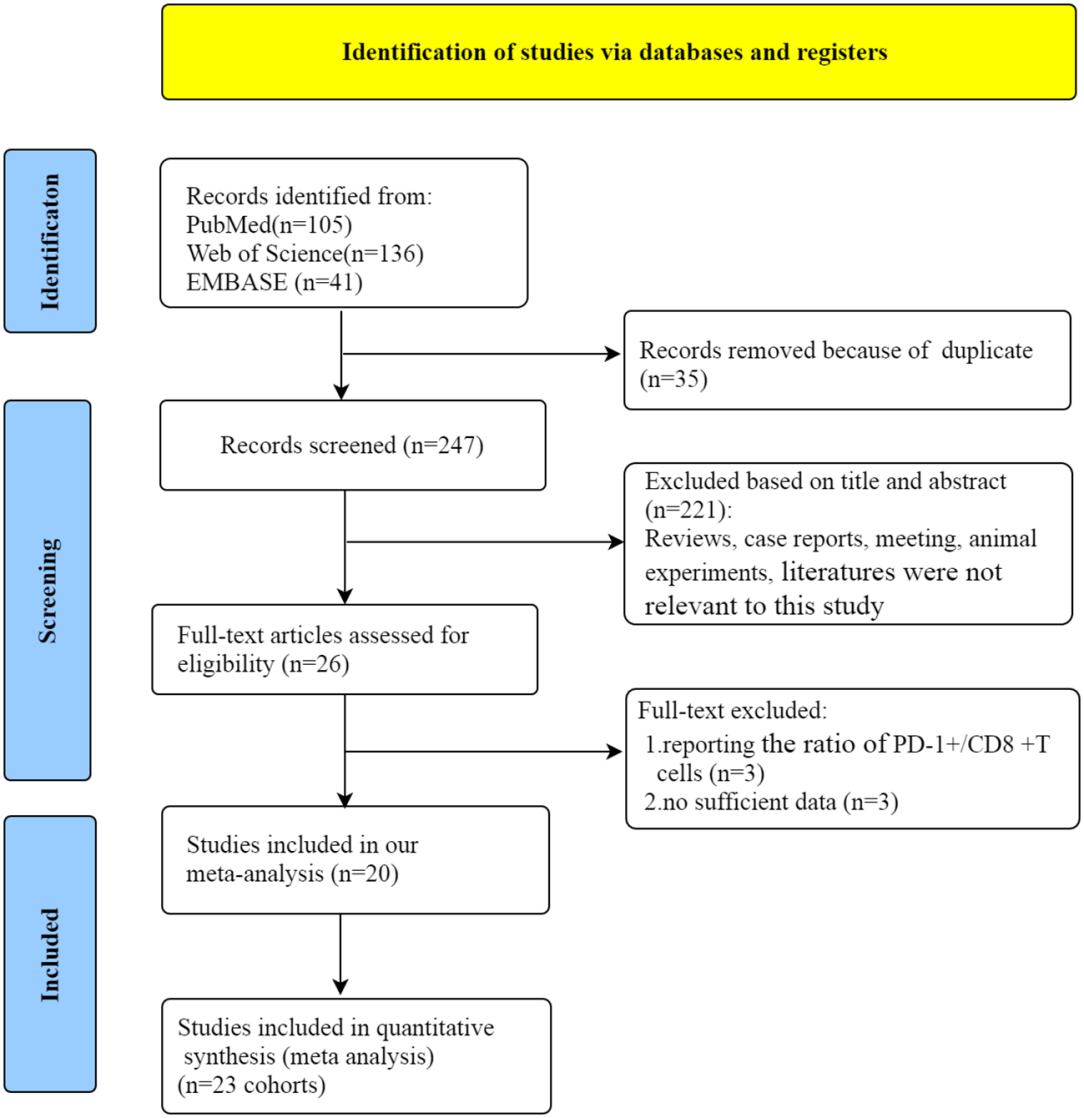
Flow chart for the literature search and study selection.

Initially, 282 articles were obtained through literature screening in PubMed (n=105), Web of Science (n=136), and Embase (n=41) databases. Of these, 35 articles were excluded due to duplication. After filtering through titles and abstracts, 224 articles were deleted for the following reasons: reviews, case reports, meeting, animal or cell-line articles and literatures were not relevant to our study. Then, a review of the entire article was conducted. Owing to report the ratio of PD-1+/CD8+ T cells and insufficient data, 6 articles were eliminated. Finally, 20 eligible studies (12, 13, 16–33) were included in our meta-analysis. The flow chart of the whole selection process is shown in **Figure 1**.

### 3.2 Study characteristics

A total of 20 studies with 3086 patients were included in our meta-analysis. They mainly come from China, Japan, South Korea, Singapore, Italy, the United States, and Switzerland, Greece. These studies included a variety of tumor types such as gastric cancer, non-small cell lung cancer, triple-negative breast cancer, pancreatic cancer, acute myeloid leukemia, hepatocellular carcinoma, head and neck cancer, mesothelioma. The specimen type of 14 articles was tissue, while 6 articles was peripheral blood. Five articles received immune checkpoint inhibitor therapy, 9 articles adopted other therapies(including surgical resection, chemotherapy, curative surgery+chemotherapy, and allo-SCT and so on) and 6 articles did not receive treatment. PD-1+CD8+ T cells was detected for protein expression by dual immunohistochemistry in 14 studies, and for flow cytometry in 6 studies. Specifically, three studies (13, 19, 20) each had two cohorts of cancer patients and then included each cohort in the quantitative analysis as an individual study. The characteristics of 20 studies are summarized in **Table 1**.

**Table 1:** Characteristics of studies included in the meta-analysis.

Author	Year	Country	Cacer type	Number	Specimen type	Treatment	Detect method	Stage	Outcome	NOS
Thommen (16)	2018	Switzerland	NSCLC	21	tissue	PD-1 blockade	Immunohistochemical double stains	advanced	OS	7
Choo (17)	2023	Singapore	gastric cancer	350	tissue	gastrectomy	mIHC/IF	all stage	OS	7
Shin (18)	2023	Korea	gastric cancer	68	peripheral blood	Chemotherapy	fluorescence-activated cell sorting	avanced	OS,PFS	7
Hsu (12)	2010	Taiwan	nasopharyngeal carcinoma	46	tissue	untreat	double immunofluorescence staining	all stage	OS,DFS	9
Yu (19)	2022	China	gastric cancer	200	tissue	Adjuvant chemotherapy	double-stained IHC	all stage	OS,DFS	9
Yu (19)	2022	China	gastric cancer	241	tissue	Adjuvant chemotherapy	double-stained IHC	all stage	OS,DFS	9
Mazzaschi (20)	2017	Italy	NSCLC	100	tissue	rescected	double immunofluorescence	all stage	OS,DFS	6
Mazzaschi (20)	2017	Italy	NSCLC	26	tissue	nivolumab	double immunofluorescence	advanced	OS	6
SAITO (21)	2019	Japan	Gastric Cancer	72	peripheral blood	untreat	multicolor flow cytometry.	all stage	OS,PFS	7
Waki (22)	2014	Japan	NSCLC	78	peripheral blood	personalized peptide vaccine	Flow cytometric	advanced	OS	6
Ma (13)	2019	China	hepatocellular carcinoma	358	tissue	untreat	multiplex immunohis-tochemistry	all stage	OS	7
Ma (13)	2019	China	hepatocellular carcinoma	254	tissue	untreat	multiplex immunohis-tochemistry	all stage	OS	7
Kansy (23)	2017	America	head and neck cancer	56	tissue	untreat	flow cytometry	advanced	DFS	6
Yang (32)	2023	China	pancreatic ductal adenocarcinoma	84	tissue	unclear	multiplex IHC	all stage	OS	7
Homicsko (31)	2023	Switzerland	mesothelioma	144	tissue	pembrolizumab	multiplex IHC	advanced	PFS	7
Yeong (30)	2019	Singapore	triple negative breast cancer	269	tissue	untreat	multiplex immunofluorescent (mIF) staining	all stage	OS,DFS	7
Shen (29)	2017	China	pancreatic cancer	94	tissue	surgical resection	double immunofuorescence staining	all stage	OS	9
Guo (27)	2020	China	triple-negative breast cancer	328	tissue	untreat	multiplexed immunohistochemistry	all stage	OS,DFS	6
You (24)	2024	Korea	acute myeloid leukaemia	60	tissue	allo-SCT	Flow cytometric analyses	all stage	OS	9
Tang (25)	2020	China	acute myeloid leukemia	50	peripheral blood	chemotherapy	multiparametric flow cytometry	all stage	OS	6
Mazzaschi (26)	2019	Italy	NSCLC	31	peripheral blood	nivolumab	Flow Mass Cytometry	advanced	OS	7
Gao (28)	2017	China	gastric adenocarcinoma	119	tissue	adjuvant chemotherapy	Immunofluorescence	all stage	OS,DFS	9
Kotsakis (33)	2019	Greece	NSCLC	37	peripheral blood	chemotherapy-naïve	Flow cytometric analyses	advanced	PFS	6

**Figure 2:**
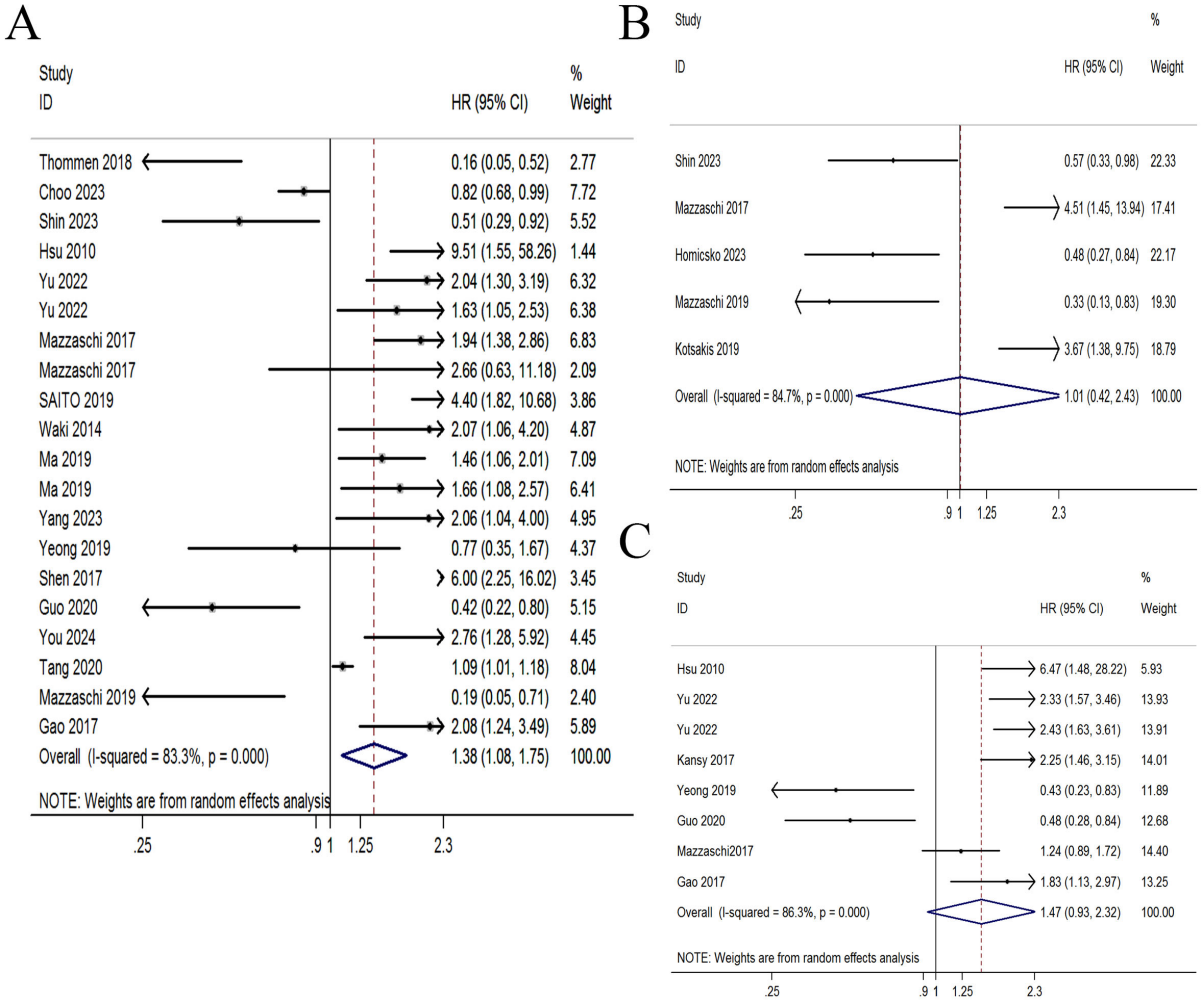
Forest plot of PD-1+CD8+ T cells with survival time. **(A)** Forest plot of PD-1+CD8+ T cells with overall survival. **(B)** Forest plot of PD-1+CD8+ T cells with progression-free survival. **(C)** Forest plot of PD-1+CD8+ T cells with disease-free survival.

Seventeen studies comprising 2849 patients evaluated the association of PD-1+CD8+ T cells with OS. An obvious heterogeneity (I^2^ = 113.51%, *p* < 0.001) was observed, so a random-effect model was applied. Patients with high PD-1+CD8+ T cells had significantly worse OS (HR = 1.379, 95% CI 1.084-1.753, *p* = 0.009, **Figure 2A**). The association between PD-1+CD8+ T cells and PFS was evaluated in 5 studies comprising 306 patients. Pooled analysis using a random-effect model demonstrated that patients with elevated PD-1+CD8+ T cells were not associated with PFS (HR = 1.006, 95% CI 0.417-2.430, *p* = 0.989, **Figure 2B**). The association between PD-1+CD8+ T cells and DFS was evaluated in 8 studies comprising 1359 patients. A random-effect model indicated that patients with elevated PD-1+CD8+ T cells had a borderline association with DFS (HR = 1.468, 95% CI 0.931-2.316, *p* = 0.099, **Figure 2C**).

### 3.3 Subgroup analyses of PD-1+CD8+ T cells in association with survival

Due to significant heterogeneity in the pooled HRs of OS in cancer patients and moderate heterogeneity in the pooled HRs of DFS in cancer patients, we conducted subgroup analysis on the selected literature to find out the sources of heterogeneity. Therefore, a subgroup analysis based on study region, cancer type, specimen type, tumor treatment, stage and detect method was carried out. The results of subgroup analyses for OS and DFS are shown in **Tables 2**, **3**, respectively.

**Table 2:** Association between PD-1+CD8+ T cells and overall survival in cancers.

				Heterogeneity		
	no of the studies	HR	95%CI	I^2^	*P*	*P*
	17	1.379	1.084-1.753	113.51	<0.0001	0.009
Area						
Asian	14	1.479	1.160-1.887	85.97	<0.0001	0.002
Europe	3	0.654	0.152-2.814	25.89	<0.0001	0.568
Cancer type						
gastric cancer	5	1.453	0.867-2.433	41.39	<0.0001	0.156
non-small cell lung cancer	4	0.868	0.325-2.324	26.88	<0.0001	0.779
Pancreatic cancer	2	3.304	1.167-9.357	3.1	0.078	0.024
triple negative breast cancer	2	0.544	0.302-0.982	1.39	0.238	0.043
acute myeloid leukaemia	2	1.6	0.654 3.914	5.58	0.018	0.303
Specimen type						
tissue	12	1.497	1.078-2.079	83.14	<0.0001	0.016
blood	5	1.08	0.559-2.088	26.29	<0.0001	0.819
Treatment						
untreat	6	1.525	0.906-2.568	28.56	<0.0001	0.112
other treatments	9	1.507	1.126-2.017	53.8	<0.0001	0.006
ICIs	3	0.421	0.063- 2.827	25.09	<0.0001	0.374
Stage						
all stage	13	1.589	1.245-2.029	83.8	<0.0001	<0.0001
advanced	5	0.626	0.225-1.738	23.82	0.001	0.368
Detect method						
double-stained IHC	12	1.384	1.079-1.776	81.96	<0.0001	0.011
Flow cytometric	4	1.662	0.607-4.547	16.02	0.001	0.323

**Table 3:** Association between PD-1+CD8+ T cells and disease-free survival in cancers.

			DFS	Heterogeneity		
	no of the studies	HR	95%CI	I2	*P*	*P*
	8	1.468	0.931-2.316	50.98	<0.0001	0.099
Asian	6	1.422	0.730-2.772	45.55	<0.0001	0.301
Europe	2	1.653	0.919-2.973	5.39	0.02	0.093
Cancer type						
gastric cancer	2	2.228	1.748-2.839	0.86	0.651	<0.0001
triple negative breast cancer	2	0.46	0.303-0.699	0.08	0.784	<0.0001
head and neck cancer	2	2.972	1.194-7.395	1.85	0.174	0.019
Treatment						
untreat	4	1.174	0.399-3.450	35.48	<0.0001	0.771
other treatments	3	1.871	1.330-2.630	8.87	0.031	<0.0001
ICIs	–	–	–	–	–	–

There was heterogeneity between research in different regions. The pooled HRs for OS in the Asian subgroup were 1.479 (95%CI: 1.160-1.887, p= 0.002), the pooled HRs for OS in the Europe subgroup were 0.654 (95%CI: 0.152-2.814, p=0.568). The pooled HRs for DFS in the Asian subgroup were 1.422 (95%CI: 0.730-2.772, p=0.301), the pooled HRs for DFS in the Europe subgroup were 1.653 (95%CI: 0.919-2.973, p=0.093). Subgroup analysis based on sample types showed that tumor tissues as sample types had poor OS (HR = 1.497, 95% CI 1.078-2.079, *p* = 0.016), while peripheral blood were not associated with OS (HR = 1.08, 95% CI 0.559-2.088, *p* = 0.819).

When the included studies were analyzed by subgroups based on cancer type. PD-1+CD8+T cells predict significantly worse OS in pancreatic cancer (HR=3.304, 95%CI 1.167-9.357, p=0.024), and significant worsening of DFS in gastric cancer and head and neck cancer (HR = 2.228, 95% CI 1.748-2.839 p<0.0001; HR = 2.972, 95% CI 1.194-7.395, p=0.019, respectively), but was associated with improved OS and DFS in triple negative breast cancer (OS: HR = 0.544, 95% CI 0.302-0.982, *p*= 0.043; DFS: HR =0.46, 95% CI 0.303-0.699, p<0.0001). No association was found between PD- L1 + CTCs and survival in other cancers. Due to the small number of studies, further studies were needed to confirm our results in the future.

When stratified for treatment, PD-1+CD8+ T cells were significantly associated with worse OS and DFS for other therapies (OS: HR = 1.507, 95% CI 1.126-2.017, *p* =0.006; DFS: HR=1.871,95%CI 1.330-2.630, p<0.0001, respectively). Surprisingly, PD-1+CD8+ T cells seemed to predict a better OS (HR = 0.421, 95% CI 0.063- 2.827, *p* = 0.374) for ICI treatment, though not reach statistically significant.

PD-1+CD8+ T cells predicted poor OS in double-stained IHC (HR = 1.384, 95% CI 1.079-1.776, *p* = 0.011). but it was not found to be related to the patient’s prognosis in flow cytometric. It is possible that there are too few studies using the same method. What’s more, patients with all stage had a worse OS(HR = 1.589, 95% CI 1.245-2.029, *p <*0.0001), while were not associated with OS in advanced patients (HR = 0.626, 95% CI 0.225-1.738, *p* =0.368).

### 3.4 Sensitivity analyses

**Figure 3:**
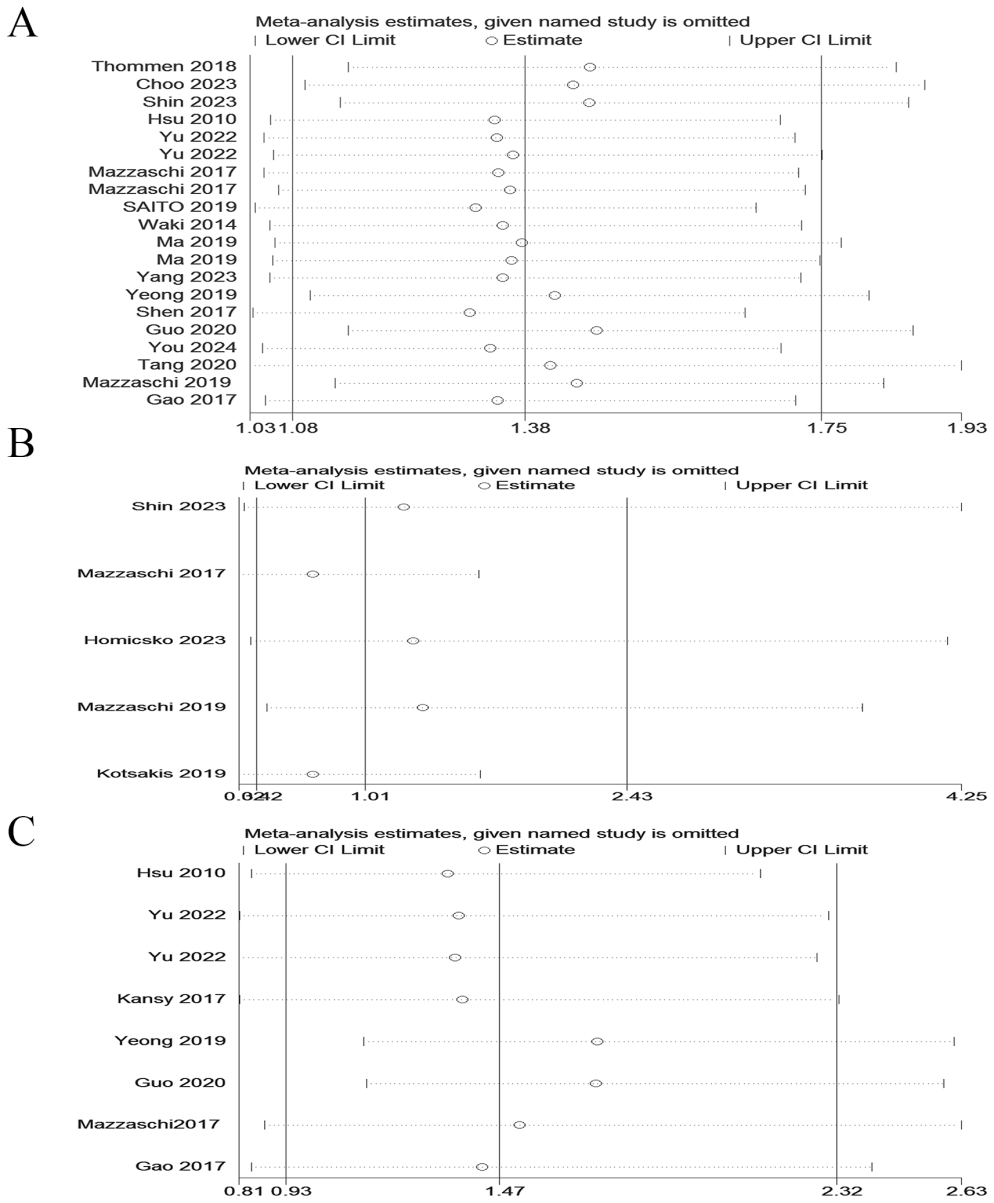
Sensitivity analyses of the pooled HRs for OS, PFS and DFS of cancer patients with PD-1+CD8+ T cells. **(A)** Sensitivity analyses of the pooled HRs for OS. **(B)** Sensitivity analyses of the pooled HRs for PFS. **(C)** Sensitivity analyses of the pooled HRs for DFS.

Due to significant heterogeneity was observed. In order to find the potential source of this heterogeneity, we carried out sensitivity analysis. Sensitivity analysis indicated that the results of our meta- analysis were robust and not significantly influenced by any single study (**Figure 3**).

### 3.5 Publication bias

**Figure 4:**
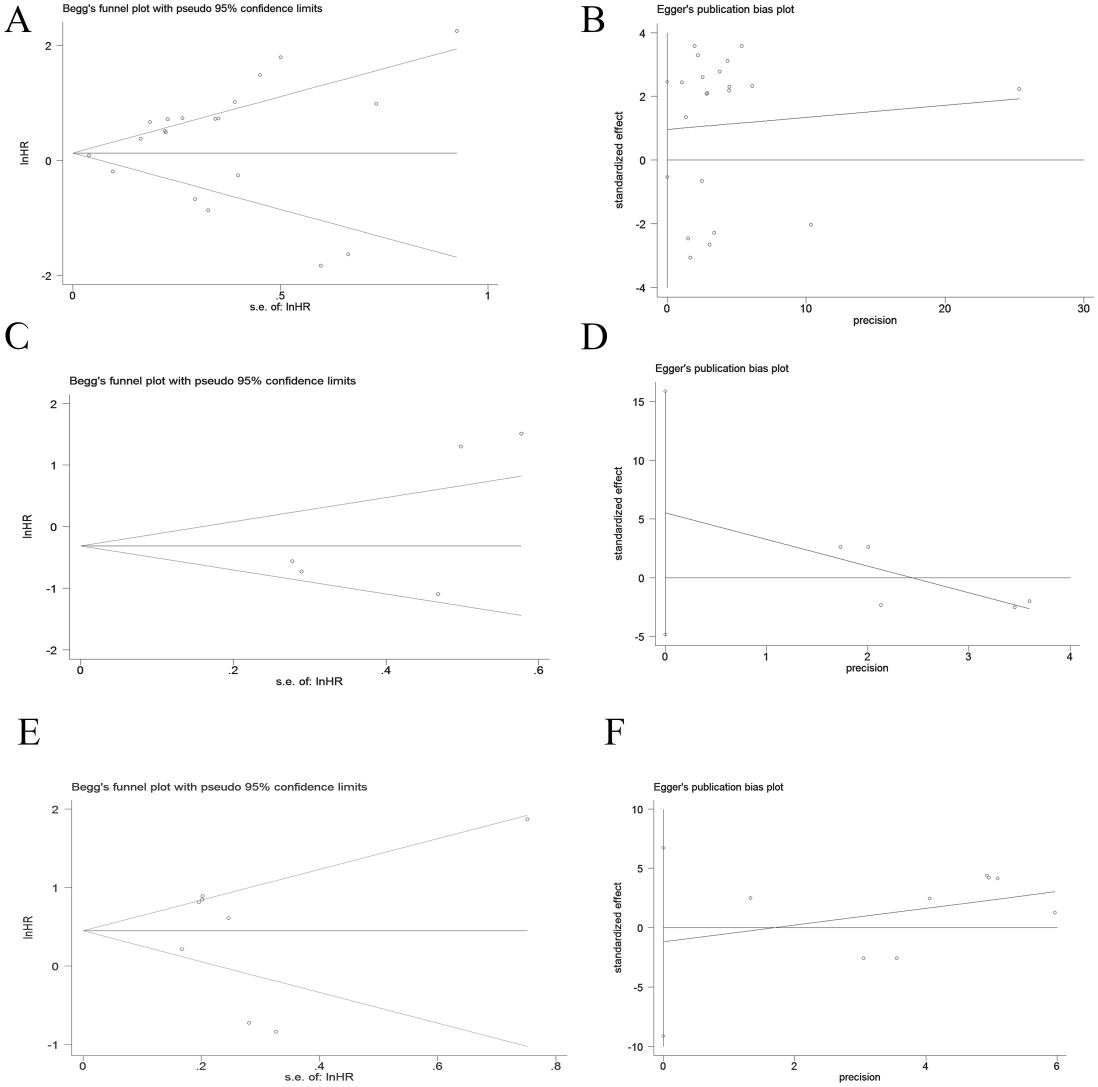
Publication bias of OS, PFS and DFS in PD-1+CD8+T cell cancer patients by Begg and Egger scatter plots. **(A, B)** Publication bias for OS. **(C, D)** Publication bias for PFS. **(E, F)** Publication bias for DFS.

Publication bias is considered the main factor affecting predictive value, so we conducted Egger and Begg tests to assess the presence of publication bias and the funnel plot symmetry was examined. Based on the shape of the funnel plot and no significant publication bias was not observed and P values in Egger and Begg tests. (p > 0.05) (**Figure 4**).

## 4 Discussion

We conducted the first meta- analysis to evaluate the clinical application of PD-1 expression on CD8+ T cells in predicting the survivals of cancer patients and to identify factors that modulate prognostic value. Overall, high PD-1 expression on CD8+ T cells is associated with worse survival outcomes, which can be potentially used as a prognostic marker of malignant tumor.

CD8+T cells that express PD-1 thought to be characterized by a state of T cell exhaustion, which is accompanied with loss the ability of cytokine production (IL-2, TNF-α and IFN-γ) and killing capacity (13, 34, 35). Recent years, researchers have studied on the relationship between the expression of PD-1 on CD8+ Tcells and the prognosis of cancer patients. Lim et al (36) indicated that higher ratio of PD-1 +/CD8 + TILs was associated with poorer overall survival, relapse-free survival and distant metastasis-free survival, besides, the ratio of PD-1 +/CD8 + TILs was the independent prognostic factor in OS, RFS and DMFS after adjusting for other significant clinicopathologic variables. However, conflicting views have been raised and no consensus has been reached. Shen et al (37) showed that PD-1 + CD8 + T cells showed equivalent function to their PD-1 - CD8 +T cells counterparts and they did not predict tumor progression in gastric cancer. Due to the discrepant results among these studies, it is difficult to apply PD-1 + CD8 + T cells to clinical applicability. Therefore, our work emphasizes the predictive value of PD-1 expression on CD8+ T cells for cancer prognosis. We reviewed 20 studies of PD-1 + CD8 + T cells, and performed a systematic meta-analysis to evaluate the expression of PD-1 on CD8+ T cells in predicting the survivals of cancer patients.

Our study indicates elevating PD-1+CD8+ T cells may predict worse survival time for cancer patients. Consistent with previous studies, researchers indicated that no matter inhibitory receptors, transcription factors or functional molecules, PD1-Hi-CD8+TIL was a exhausted T cell, thus promoting tumor growth, avoiding immune surveillance and promoting immune escape. However, significant heterogeneity was observed in our study. To find the source of heterogeneity, we analyzed the factors that may affect heterogeneity. we stratified the meta-analysis by cancer type and found PD-1+CD8+ T cells predicted significantly worse OS in pancreatic cancer and poor DFS in gastric cancer and head and neck cancer while it was associated with improved OS and DFS in triple negative breast cancer. This may be due to the heterogeneity of PD-1 CD8+ T cells in their roles in different tumors. Odorizzi et al. (38) demonstrated that T cells can be differentiated to reach terminal exhaustion in the genetic absence of PD-1. Moreover, a recent breast cancer study also revealed that there is no significant reduction in cytokine production in PD-1 + T cells compared with PD-1- T cells (39). The immune microenvironment in TNBC may not be as suppressed as in other tumors. However, the exact mechanism of PD-1 CD8+ T cells in triple negative cancer requires further research.

PD-1 is a cytotoxic T cell immune checkpoint receptor that has inhibitory effects when bound to its ligand(PD-L1). The use of checkpoint blocking antibodies to block PD-1 immunotherapy can restore and enhance the response of cytotoxic T cells to chemotherapy resistant tumors, resulting in sustained response and tolerable toxicity, and prolonging overall survival. Studies have shown that PD-L1 expression responds better to ICI treatment and have a longer survival period in patients receiving immune checkpoint inhibitor therapy (40, 41). Due to this mechanism, the PD-L1/PD-1 axis has been found to be an crucial mechanism by which tumor cells evade T cell immunity, PD-1 inhibitors revitalize CD8+T cells by blocking the PD-1/PD-L1 pathway, promoting their proliferation and functional recovery (42–44). In addition, PD-1/PD-L1 axis inhibitors disrupt the interaction between PD-1 and PD-L1 and subsequently restore the immune response to tumor cells, ultimately improving survival outcomes for cancer patients (45). Homicsko et al (31) pointed out that PD-1 expression of CD8 T cells is an independent predictive factor for the clinical benefits of PD-1 inhibition in patients with advanced mesothelioma. In advanced NSCLC treated with anti-PD1 therapy, Mazzaschi et al (26) found high circulating PD-1+CD8+ cells provided a significantly prolonged progression-free survival. While some studies did not find a significant association between PD-1+CD8+ and survival for ICI treatment (20). Subgroup meta-analysis that gathered these studies showed that patients with high levels of PD-1+CD8+T cells and treated with PD- 1/PD- L1 inhibitors may have prolonged OS, though it not reach the significance. Nevertheless, the expression of PD-1+ CD8+ T cells is a potential prognostic marker for ICI treatment, which needs to be verified by larger studies in the future. More importantly, compared to ICI treatment, our meta-analysis showed a significant association between PD-1+ CD8+T cells and survival in patients receiving non-ICI treatment, with significantly shorter overall survival in patients with PD-1+ CD8+T cells.

According to the above analysis, high PD-1 expression on CD8+ T cells had a adverse survival time, which can be potentially used as a prognostic marker of malignant tumor. However, some limitations are exist in our study. First, although we tried to collect all the articles, some data were lost because our study was restricted to articles published only in English or Chinese, and it is hoped that future research efforts will include publications in other languages to ensure a more comprehensive analysis. Second, there was obvious heterogeneity in our study, which may be caused by the study region, cancer type, treatments, specimen type and detect method. Third, most of the included studies had very small sample sizes. More large-scale studies with PD-1expression on CD8+ T cells are needed in the future to validate the findings of our meta-analysis.

## 5 Conclusion

Our analysis demonstrated that high PD-1 expression on CD8+ T cells had a adverse survival time, which could be potentially used as a prognostic marker of malignant tumor.
