# Supplementary material for: Prognostic significance of programmed cell death 1 expression on CD8+T cells in various cancers: a systematic review and meta-analysis
Source: Front Oncol. 2025 Jan 14;14:1531219. doi: 10.3389/fonc.2024.1531219 (PMC11772205; doi:10.3389/fonc.2024.1531219)
Supplement: Supplementary file 1 [file Table1.docx]

**Supplementary Table 1.** Summary of lipids, chronic kidney disease, estimated glomerular filtration rate and blood urea Nitrogen.

| Category | Variable | Population | Number of SNPs | Sample Size | Year | Consortium | GWAS ID |
| --- | --- | --- | --- | --- | --- | --- | --- |
| Lipids | LDL-C | European | 12,321,875 | 440,546 | 2020 | UK Biobank | ieu-b-110 |
|  | Triglycerides | European | 12,321,875 | 441,016 | 2020 | UK Biobank | ieu-b-111 |
| Primary outcome | Chronic kidney disease | European | - | 480,698 | 2019 | CKDGen | - |
|  | Chronic kidney disease | European | 2,179,497 | 117,165 | 2016 | Ebi | ebi-a-GCST003374 |
|  | Chronic kidney disease | European | 16,380,459 | 216,743 | 2021 | FinnGen | finn-b-N14_CHRONKIDNEYDIS |
| Secondary outcome | eGFR | European | - | 480,698 | 2019 | CKDGen | - |
|  | BUN | European | - | 480,698 | 2019 | CKDGen | - |
| Positive control | Coronary heart disease | European | 9,455,779 | 184,305 | 2015 | CARDIoGRAMplusC4D | ieu-a-7 |
| Mediation | Immune cell | European | 15,030,660 | 3,618 | 2020 | Ebi | ebi-a-GCST90002007 |

**Supplementary Table 2.** The detail of instrumental variable corresponding to LDLR.

| chr.exposure | pos.exposure | beta.exposure | se.exposure | pval.exposure | SNP | effect_allele.exposure | other_allele.exposure | eaf.exposure |
| --- | --- | --- | --- | --- | --- | --- | --- | --- |
| 19 | 11164968 | -0.0134092 | 0.00245241 | 4.60E-08 | rs2421198 | T | C | 0.232896 |
| 19 | 11174441 | -0.0236774 | 0.00419212 | 1.60E-08 | rs73013196 | T | C | 0.067976 |
| 19 | 11190764 | 0.0427212 | 0.00402001 | 2.20E-26 | rs36005514 | A | G | 0.071349 |
| 19 | 11284302 | -0.0189721 | 0.00206993 | 4.90E-20 | rs379309 | T | C | 0.501714 |
| 19 | 11302606 | 0.016716 | 0.00208789 | 1.20E-15 | rs7250652 | G | A | 0.443277 |
| 19 | 11146499 | -0.0305197 | 0.00208477 | 1.60E-48 | rs3786721 | C | T | 0.557713 |
| 19 | 11147526 | -0.17308 | 0.00989319 | 1.60E-68 | rs73013176 | C | T | 0.011456 |
| 19 | 11158047 | -0.0165393 | 0.00235425 | 2.10E-12 | rs10420325 | T | A | 0.639284 |
| 19 | 11169305 | 0.0360512 | 0.00527004 | 7.90E-12 | rs36049922 | C | T | 0.040127 |
| 19 | 11257018 | 0.0141311 | 0.0023743 | 2.70E-09 | rs11557092 | C | T | 0.735703 |
| 19 | 11264618 | 0.0455464 | 0.00547214 | 8.60E-17 | rs140181075 | T | C | 0.037619 |
| 19 | 11266693 | 0.0416562 | 0.00225634 | 4.20E-76 | rs7251031 | G | T | 0.306748 |
| 19 | 11267940 | -0.0316003 | 0.0025504 | 2.90E-35 | rs11673593 | T | A | 0.790703 |
| 19 | 11269893 | -0.0572931 | 0.00211261 | 5.79E-162 | rs76213248 | T | C | 0.410251 |
| 19 | 11123091 | 0.0239223 | 0.0040324 | 3.00E-09 | rs117819913 | A | T | 0.07124 |
| 19 | 11126160 | -0.0889437 | 0.00415804 | 1.60E-101 | rs73013166 | C | T | 0.066524 |
| 19 | 11133827 | 0.0346014 | 0.00290269 | 9.30E-33 | rs12609589 | T | C | 0.153388 |
| 19 | 11152888 | -0.0437918 | 0.00432384 | 4.20E-24 | rs144624209 | A | G | 0.064106 |
| 19 | 11158055 | 0.0265587 | 0.00399608 | 3.00E-11 | rs4804564 | T | C | 0.927533 |
| 19 | 11183837 | -0.0576403 | 0.00241122 | 2.70E-126 | rs73015007 | A | G | 0.250898 |
| 19 | 11185919 | -0.103305 | 0.00270769 | 1.00E-200 | rs10423733 | C | T | 0.180142 |
| 19 | 11204627 | 0.0315779 | 0.00420565 | 6.00E-14 | rs17242367 | T | C | 0.070321 |
| 19 | 11207516 | -0.112139 | 0.00553933 | 4.00E-91 | rs73015030 | A | G | 0.036317 |
| 19 | 11227480 | 0.0422734 | 0.00211024 | 2.90E-89 | rs2738447 | C | A | 0.592612 |
| 19 | 11230690 | 0.0681552 | 0.00524329 | 1.20E-38 | rs116959285 | G | C | 0.045717 |
| 19 | 11235423 | 0.0239253 | 0.00216633 | 2.30E-28 | rs6511724 | C | T | 0.642857 |
| 19 | 11277922 | 0.029107 | 0.00481642 | 1.50E-09 | rs111731690 | T | G | 0.05169 |
| 19 | 11284028 | 0.0153178 | 0.00229125 | 2.30E-11 | rs4804149 | C | T | 0.287226 |
| 19 | 11335477 | -0.0450814 | 0.00559272 | 7.60E-16 | rs8101801 | A | C | 0.035559 |
| 19 | 11116266 | 0.0192288 | 0.00331813 | 6.80E-09 | rs13345127 | T | C | 0.109899 |
| 19 | 11127139 | -0.048528 | 0.00677934 | 8.20E-13 | rs117234045 | A | G | 0.025203 |
| 19 | 11206040 | -0.0656844 | 0.00830137 | 2.50E-15 | rs17248748 | T | C | 0.015825 |
| 19 | 11206575 | -0.0474014 | 0.00207343 | 1.10E-115 | rs6511721 | A | G | 0.519695 |
| 19 | 11241428 | 0.0531066 | 0.00378972 | 1.30E-44 | rs17249001 | A | G | 0.088845 |
| 19 | 11242307 | 0.0257528 | 0.00317135 | 4.60E-16 | rs2738464 | C | G | 0.87843 |
| 19 | 11248104 | -0.0710448 | 0.00631556 | 2.30E-29 | rs147540853 | A | G | 0.027789 |
| 19 | 11257169 | -0.0704358 | 0.00264596 | 4.00E-156 | rs79668907 | T | C | 0.228499 |
| 19 | 11176337 | 0.0451213 | 0.00470452 | 8.70E-22 | rs11671812 | T | C | 0.051222 |
| 19 | 11184250 | -0.0486439 | 0.00210973 | 1.30E-117 | rs2421201 | G | A | 0.501212 |
| 19 | 11192603 | -0.14759 | 0.00830891 | 1.40E-70 | rs112159161 | T | C | 0.016621 |
| 19 | 11211077 | 0.0414739 | 0.00433606 | 1.10E-21 | rs3745677 | A | G | 0.061743 |
| 19 | 11257499 | 0.0450148 | 0.00694567 | 9.10E-11 | rs141618758 | C | T | 0.024108 |
| 19 | 11282298 | -0.060965 | 0.00817716 | 9.00E-14 | rs117339792 | A | G | 0.01984 |
| 19 | 11283222 | -0.107272 | 0.00482702 | 2.00E-109 | rs146576912 | T | C | 0.060983 |
| 19 | 11336626 | -0.0201813 | 0.00367256 | 3.90E-08 | rs1433091 | G | A | 0.906073 |

**Supplementary Table 3.** The detail of instrumental variable corresponding to HMGCR.

| chr.exposure | pos.exposure | beta.exposure | se.exposure | pval.exposure | SNP | effect_allele.exposure | other_allele.exposure | eaf.exposure |
| --- | --- | --- | --- | --- | --- | --- | --- | --- |
| 5 | 74624484 | -0.0372115 | 0.00487202 | 2.20E-14 | rs75240579 | T | C | 0.048363 |
| 5 | 74562029 | 0.03556 | 0.00261075 | 3.00E-42 | rs2006760 | G | C | 0.205486 |
| 5 | 74664987 | -0.0271295 | 0.00433093 | 3.70E-10 | rs62366588 | A | C | 0.065948 |
| 5 | 74615209 | 0.0532822 | 0.00653971 | 3.70E-16 | rs141642272 | C | G | 0.026705 |
| 5 | 74651864 | 0.042154 | 0.0029315 | 6.90E-47 | rs55727654 | A | G | 0.14871 |
| 5 | 74623949 | 0.0243909 | 0.00372718 | 6.00E-11 | rs111353455 | A | G | 0.085844 |
| 5 | 74641707 | 0.0333589 | 0.00345272 | 4.40E-22 | rs2303152 | A | G | 0.101442 |
| 5 | 74729433 | -0.0303618 | 0.00499242 | 1.20E-09 | rs116153450 | A | C | 0.04623 |
| 5 | 74656539 | 0.0621175 | 0.00212705 | 1.70E-187 | rs12916 | C | T | 0.400537 |
| 5 | 74682474 | 0.0394972 | 0.00635898 | 5.30E-10 | rs17562727 | C | T | 0.027617 |
| 5 | 74717761 | -0.0260509 | 0.00385694 | 1.40E-11 | rs80324692 | T | C | 0.081157 |
| 5 | 74563700 | 0.048608 | 0.00785612 | 6.10E-10 | rs115845757 | A | G | 0.019 |
| 5 | 74650106 | 0.0619849 | 0.00619367 | 1.40E-23 | rs17648121 | T | C | 0.029877 |
| 5 | 74682600 | 0.0329927 | 0.00582168 | 1.50E-08 | rs140092661 | T | A | 0.034128 |
| 5 | 74757657 | 0.0251785 | 0.00459392 | 4.20E-08 | rs12659331 | C | A | 0.054326 |
| 5 | 74630829 | 0.0564278 | 0.00316653 | 4.90E-71 | rs72633963 | A | G | 0.1238 |
| 5 | 74560487 | 0.0410063 | 0.00216518 | 5.40E-80 | rs10051965 | T | C | 0.369925 |
| 5 | 74610293 | -0.0281057 | 0.00423755 | 3.30E-11 | rs35122945 | C | A | 0.067368 |
| 5 | 74602898 | 0.0244938 | 0.00297742 | 1.90E-16 | rs4703665 | C | T | 0.848709 |

**Supplementary Table 4.** The detail of instrumental variable corresponding to PCSK9.

| chr.exposure | pos.exposure | beta.exposure | se.exposure | pval.exposure | SNP | effect_allele.exposure | other_allele.exposure | eaf.exposure |
| --- | --- | --- | --- | --- | --- | --- | --- | --- |
| 1 | 55433978 | -0.0234719 | 0.00358388 | 5.80E-11 | rs6691964 | A | G | 0.092472 |
| 1 | 55491135 | 0.0175746 | 0.00243048 | 4.80E-13 | rs556369867 | T | C | 0.330585 |
| 1 | 55494301 | -0.0334061 | 0.00501479 | 2.70E-11 | rs72909541 | T | C | 0.046097 |
| 1 | 55520938 | 0.0452728 | 0.00520209 | 3.20E-18 | rs150119739 | A | G | 0.045318 |
| 1 | 55522558 | 0.0454642 | 0.0075822 | 2.00E-09 | rs7525503 | T | G | 0.02036 |
| 1 | 55522674 | -0.0282322 | 0.00279415 | 5.30E-24 | rs11587071 | T | C | 0.168881 |
| 1 | 55538552 | -0.0531381 | 0.00394676 | 2.60E-41 | rs10493176 | G | T | 0.07579 |
| 1 | 55489960 | -0.0297494 | 0.00231882 | 1.10E-37 | rs3976734 | G | A | 0.374504 |
| 1 | 55491853 | -0.0543492 | 0.00278155 | 5.10E-85 | rs200730299 | C | A | 0.195034 |
| 1 | 55496131 | 0.0305717 | 0.00365832 | 6.40E-17 | rs17192725 | A | G | 0.095408 |
| 1 | 55503448 | 0.0406795 | 0.00235743 | 1.00E-66 | rs17111503 | G | A | 0.268141 |
| 1 | 55516713 | -0.0168117 | 0.00295297 | 1.20E-08 | rs7546522 | T | C | 0.155442 |
| 1 | 55518316 | -0.0295845 | 0.00214514 | 2.90E-43 | rs2483205 | T | C | 0.438633 |
| 1 | 55551718 | 0.0314531 | 0.00517068 | 1.20E-09 | rs11583974 | A | G | 0.042146 |
| 1 | 55576102 | -0.0475957 | 0.00671909 | 1.40E-12 | rs56349475 | C | T | 0.024601 |
| 1 | 55588142 | -0.0336489 | 0.00562029 | 2.10E-09 | rs79396670 | A | G | 0.035496 |
| 1 | 55453841 | -0.0538858 | 0.00722418 | 8.70E-14 | rs146273942 | A | G | 0.023188 |
| 1 | 55492190 | -0.0283879 | 0.0023826 | 9.90E-33 | rs2479420 | T | C | 0.73803 |
| 1 | 55496861 | -0.0294547 | 0.00507333 | 6.40E-09 | rs11810371 | A | G | 0.043743 |
| 1 | 55505647 | -0.348456 | 0.00793088 | 1.00E-200 | rs11591147 | T | G | 0.017468 |
| 1 | 55507649 | 0.0316517 | 0.0021463 | 3.20E-49 | rs11206513 | T | C | 0.600617 |
| 1 | 55526428 | 0.0680285 | 0.00580615 | 1.00E-31 | rs11206517 | G | T | 0.033149 |
| 1 | 55448842 | 0.0177548 | 0.0025792 | 5.80E-12 | rs2495517 | G | A | 0.794271 |
| 1 | 55470153 | -0.10344 | 0.0073736 | 1.00E-44 | rs12732125 | T | C | 0.020368 |
| 1 | 55484582 | 0.0125674 | 0.00221762 | 1.50E-08 | rs2479395 | C | T | 0.668453 |
| 1 | 55485042 | 0.0481535 | 0.00605559 | 1.80E-15 | rs77875082 | A | G | 0.032388 |
| 1 | 55513183 | -0.0386615 | 0.00705365 | 4.20E-08 | rs41294821 | T | C | 0.022807 |
| 1 | 55521313 | 0.0425743 | 0.00218093 | 7.30E-85 | rs472495 | T | G | 0.648959 |
| 1 | 55583210 | -0.192336 | 0.00997554 | 7.80E-83 | rs530804537 | A | G | 0.011303 |
| 1 | 55466303 | -0.0187129 | 0.00324835 | 8.40E-09 | rs55637835 | T | C | 0.120881 |
| 1 | 55496648 | -0.0202563 | 0.00254032 | 1.50E-15 | rs12739979 | T | C | 0.246521 |
| 1 | 55500978 | 0.0509816 | 0.00777535 | 5.50E-11 | rs72660548 | G | C | 0.018458 |
| 1 | 55518622 | -0.0340702 | 0.0048672 | 2.60E-12 | rs45613943 | C | T | 0.048725 |

**Supplementary Table 5.** The detail of instrumental variable corresponding to NPC1L1.

| chr.exposure | pos.exposure | beta.exposure | se.exposure | pval.exposure | id.exposure | SNP | effect_allele.exposure | other_allele.exposure |
| --- | --- | --- | --- | --- | --- | --- | --- | --- |
| 7 | 44592091 | -0.0213142 | 0.00211012 | 5.50E-24 | ieu-b-110 | rs217399 | T | C |
| 7 | 44596644 | 0.0259282 | 0.00392582 | 4.00E-11 | ieu-b-110 | rs73107478 | C | A |
| 7 | 44570067 | -0.0133498 | 0.00234076 | 1.20E-08 | ieu-b-110 | rs11763759 | C | T |
| 7 | 44582331 | 0.0355498 | 0.00267287 | 2.30E-40 | ieu-b-110 | rs2073547 | G | A |
| 7 | 44559803 | 0.017365 | 0.00256025 | 1.20E-11 | ieu-b-110 | rs148825701 | T | C |
| 7 | 44586578 | 0.0249436 | 0.00363876 | 7.10E-12 | ieu-b-110 | rs12666108 | C | T |

**Supplementary Table 6.** The detail of instrumental variable corresponding to APOB.

| chr.exposure | pos.exposure | beta.exposure | se.exposure | pval.exposure | SNP | effect_allele.exposure | other_allele.exposure | eaf.exposure |
| --- | --- | --- | --- | --- | --- | --- | --- | --- |
| 2 | 21147179 | 0.0598493 | 0.0032162 | 2.70E-77 | rs4128553 | T | C | 0.119564 |
| 2 | 21208211 | -0.0527285 | 0.0024772 | 1.50E-100 | rs7557067 | G | A | 0.227783 |
| 2 | 21246382 | -0.0729131 | 0.00986922 | 1.50E-13 | rs72653066 | G | C | 0.011361 |
| 2 | 21250223 | 0.0746047 | 0.00209394 | 1.00E-200 | rs11901649 | A | G | 0.443659 |
| 2 | 21334717 | -0.0959288 | 0.0099138 | 3.80E-22 | rs114185526 | T | C | 0.011161 |
| 2 | 21349199 | -0.0189781 | 0.0021617 | 1.60E-18 | rs312961 | C | T | 0.366218 |
| 2 | 21366682 | -0.0816866 | 0.00923295 | 9.00E-19 | rs72782175 | C | T | 0.012867 |
| 2 | 21135220 | 0.0709235 | 0.0100731 | 1.90E-12 | rs137978266 | G | A | 0.011587 |
| 2 | 21182116 | -0.0809843 | 0.00417288 | 6.70E-84 | rs36134738 | G | A | 0.069376 |
| 2 | 21216112 | -0.0214817 | 0.00380881 | 1.70E-08 | rs76384951 | C | A | 0.082516 |
| 2 | 21238897 | 0.0749277 | 0.00751954 | 2.20E-23 | rs497166 | C | T | 0.980477 |
| 2 | 21261998 | 0.04341 | 0.0074869 | 6.70E-09 | rs12720796 | C | A | 0.019689 |
| 2 | 21133883 | -0.0465695 | 0.00338712 | 5.20E-43 | rs10198175 | G | A | 0.891006 |
| 2 | 21149771 | -0.0364752 | 0.00219716 | 6.80E-62 | rs140798831 | C | T | 0.656841 |
| 2 | 21179379 | -0.0709265 | 0.00960711 | 1.60E-13 | rs149196850 | A | G | 0.012092 |
| 2 | 21241505 | -0.064958 | 0.00364692 | 5.70E-71 | rs12713956 | G | A | 0.089244 |
| 2 | 21289068 | -0.0804294 | 0.00502529 | 1.20E-57 | rs72902590 | A | G | 0.044841 |
| 2 | 21323839 | 0.0795386 | 0.00374364 | 3.60E-100 | rs377122620 | G | T | 0.112914 |
| 2 | 21205563 | 0.0282968 | 0.0022359 | 1.00E-36 | rs10164442 | G | A | 0.31836 |
| 2 | 21228827 | 0.0394744 | 0.00348176 | 8.60E-30 | rs1801701 | T | C | 0.098843 |
| 2 | 21229446 | 0.0981243 | 0.0104207 | 4.70E-21 | rs1042023 | C | G | 0.010503 |
| 2 | 21297051 | 0.0764916 | 0.00221254 | 1.00E-200 | rs1897083 | G | A | 0.334676 |
| 2 | 21301892 | 0.046023 | 0.00493809 | 1.20E-20 | rs6756743 | T | C | 0.047286 |
| 2 | 21145665 | -0.0442269 | 0.00563616 | 4.30E-15 | rs10175646 | C | T | 0.964662 |
| 2 | 21216815 | 0.065847 | 0.00215559 | 1.00E-200 | rs62122481 | A | C | 0.376939 |
| 2 | 21220955 | -0.0487348 | 0.00260358 | 3.50E-78 | rs4665709 | A | G | 0.19872 |
| 2 | 21233972 | -0.115228 | 0.00533769 | 2.30E-103 | rs533617 | C | T | 0.039479 |
| 2 | 21253628 | 0.0628605 | 0.0032695 | 2.20E-82 | rs12720807 | T | A | 0.118098 |
| 2 | 21257927 | 0.0618146 | 0.00553677 | 6.10E-29 | rs12720842 | C | T | 0.03649 |
| 2 | 21263639 | 0.0989323 | 0.00293061 | 1.00E-200 | rs531819 | G | T | 0.850253 |
| 2 | 21295065 | -0.0283419 | 0.00304812 | 1.40E-20 | rs540156 | T | C | 0.140353 |

**Supplementary Table 7.** The detail of instrumental variable corresponding to ABCG5.

| chr.exposure | pos.exposure | beta.exposure | se.exposure | pval.exposure | SNP | effect_allele.exposure | other_allele.exposure | eaf.exposure |
| --- | --- | --- | --- | --- | --- | --- | --- | --- |
| 2 | 44044885 | 0.023052 | 0.0029703 | 8.40E-15 | rs139029940 | A | C | 0.149793 |
| 2 | 44069772 | -0.0991016 | 0.0042053 | 8.61E-123 | rs75331444 | A | G | 0.065626 |
| 2 | 44077225 | -0.0466276 | 0.00400593 | 2.60E-31 | rs7590687 | C | T | 0.923041 |
| 2 | 44125058 | 0.0192904 | 0.0030037 | 1.30E-10 | rs59867553 | T | C | 0.140592 |
| 2 | 44044839 | -0.0406493 | 0.00409378 | 3.10E-23 | rs13427362 | G | A | 0.069877 |
| 2 | 44094845 | -0.087527 | 0.00860213 | 2.60E-24 | rs6733452 | A | G | 0.015445 |
| 2 | 44009142 | -0.0883053 | 0.0125314 | 1.80E-12 | rs576115488 | G | T | 0.010006 |
| 2 | 44038697 | -0.0154358 | 0.00270841 | 1.20E-08 | rs10180615 | C | G | 0.182461 |
| 2 | 44049120 | 0.0192523 | 0.00242516 | 2.00E-15 | rs4245786 | A | G | 0.757358 |
| 2 | 44073881 | -0.0537152 | 0.00222274 | 5.00E-129 | rs6544713 | C | T | 0.676864 |
| 2 | 44075217 | 0.0432792 | 0.00440787 | 9.40E-23 | rs17424122 | A | T | 0.06197 |
| 2 | 44100063 | 0.0347481 | 0.00484782 | 7.60E-13 | rs72798839 | T | C | 0.048328 |
| 2 | 44055527 | -0.0287298 | 0.00209558 | 8.90E-43 | rs4549146 | C | T | 0.448266 |
| 2 | 44063346 | 0.0490825 | 0.00509965 | 6.30E-22 | rs140488605 | T | C | 0.04897 |
| 2 | 44084975 | -0.0359316 | 0.00525162 | 7.80E-12 | rs72796772 | T | C | 0.043263 |
| 2 | 44101394 | -0.0165085 | 0.00238761 | 4.70E-12 | rs4953027 | G | A | 0.744955 |
| 2 | 43944191 | -0.0637139 | 0.0102737 | 5.60E-10 | rs142761319 | T | G | 0.011085 |
| 2 | 44077323 | -0.104551 | 0.00800801 | 5.90E-39 | rs77673991 | T | C | 0.017381 |
| 2 | 44079401 | -0.024704 | 0.00445397 | 2.90E-08 | rs56260466 | A | G | 0.059992 |
| 2 | 44093276 | -0.0489328 | 0.00256145 | 2.40E-81 | rs7598542 | C | G | 0.210257 |

**Supplementary Table 8.** The detail of instrumental variable corresponding to ABCG8.

| chr.exposure | pos.exposure | beta.exposure | se.exposure | pval.exposure | SNP | effect_allele.exposure | other_allele.exposure | eaf.exposure |
| --- | --- | --- | --- | --- | --- | --- | --- | --- |
| 2 | 44044885 | 0.023052 | 0.0029703 | 8.40E-15 | rs139029940 | A | C | 0.149793 |
| 2 | 44069772 | -0.0991016 | 0.0042053 | 8.61E-123 | rs75331444 | A | G | 0.065626 |
| 2 | 44077225 | -0.0466276 | 0.00400593 | 2.60E-31 | rs7590687 | C | T | 0.923041 |
| 2 | 44125058 | 0.0192904 | 0.0030037 | 1.30E-10 | rs59867553 | T | C | 0.140592 |
| 2 | 44044839 | -0.0406493 | 0.00409378 | 3.10E-23 | rs13427362 | G | A | 0.069877 |
| 2 | 44094845 | -0.087527 | 0.00860213 | 2.60E-24 | rs6733452 | A | G | 0.015445 |
| 2 | 44009142 | -0.0883053 | 0.0125314 | 1.80E-12 | rs576115488 | G | T | 0.010006 |
| 2 | 44038697 | -0.0154358 | 0.00270841 | 1.20E-08 | rs10180615 | C | G | 0.182461 |
| 2 | 44049120 | 0.0192523 | 0.00242516 | 2.00E-15 | rs4245786 | A | G | 0.757358 |
| 2 | 44073881 | -0.0537152 | 0.00222274 | 5.00E-129 | rs6544713 | C | T | 0.676864 |
| 2 | 44075217 | 0.0432792 | 0.00440787 | 9.40E-23 | rs17424122 | A | T | 0.06197 |
| 2 | 44100063 | 0.0347481 | 0.00484782 | 7.60E-13 | rs72798839 | T | C | 0.048328 |
| 2 | 44055527 | -0.0287298 | 0.00209558 | 8.90E-43 | rs4549146 | C | T | 0.448266 |
| 2 | 44063346 | 0.0490825 | 0.00509965 | 6.30E-22 | rs140488605 | T | C | 0.04897 |
| 2 | 44084975 | -0.0359316 | 0.00525162 | 7.80E-12 | rs72796772 | T | C | 0.043263 |
| 2 | 44101394 | -0.0165085 | 0.00238761 | 4.70E-12 | rs4953027 | G | A | 0.744955 |
| 2 | 44077323 | -0.104551 | 0.00800801 | 5.90E-39 | rs77673991 | T | C | 0.017381 |
| 2 | 44079401 | -0.024704 | 0.00445397 | 2.90E-08 | rs56260466 | A | G | 0.059992 |
| 2 | 44093276 | -0.0489328 | 0.00256145 | 2.40E-81 | rs7598542 | C | G | 0.210257 |

**Supplementary Table 9.** The detail of instrumental variable corresponding to LPL.

| chr.exposure | pos.exposure | beta.exposure | se.exposure | pval.exposure | SNP | effect_allele.exposure | other_allele.exposure | eaf.exposure |
| --- | --- | --- | --- | --- | --- | --- | --- | --- |
| 8 | 19723503 | 0.0214263 | 0.00212817 | 7.70E-24 | rs2044061 | C | T | 0.321186 |
| 8 | 19727047 | 0.0584298 | 0.0027247 | 5.11E-102 | rs1441778 | T | C | 0.843832 |
| 8 | 19729605 | -0.025995 | 0.00248125 | 1.10E-25 | rs1441779 | C | T | 0.796007 |
| 8 | 19749390 | -0.0310554 | 0.00198677 | 4.50E-55 | rs3898938 | T | C | 0.48184 |
| 8 | 19768150 | -0.149125 | 0.00841587 | 3.00E-70 | rs142084074 | A | G | 0.015151 |
| 8 | 19813676 | 0.0250664 | 0.00277053 | 1.50E-19 | rs270 | A | C | 0.158181 |
| 8 | 19815098 | 0.0432872 | 0.0025137 | 1.90E-66 | rs283 | T | C | 0.199272 |
| 8 | 19815556 | -0.116913 | 0.00229945 | 1.00E-200 | rs287 | G | A | 0.246649 |
| 8 | 19848117 | 0.0757662 | 0.0083203 | 8.50E-20 | rs11781692 | A | C | 0.014572 |
| 8 | 19852491 | 0.0720911 | 0.00674999 | 1.30E-26 | rs74444445 | C | T | 0.023877 |
| 8 | 19888586 | -0.194709 | 0.00538679 | 1.00E-200 | rs138295898 | C | T | 0.037021 |
| 8 | 19890641 | 0.0527234 | 0.00792251 | 2.80E-11 | rs187544997 | G | C | 0.016545 |
| 8 | 19903238 | 0.0518802 | 0.00786956 | 4.30E-11 | rs144469617 | A | G | 0.016219 |
| 8 | 19918088 | -0.142708 | 0.0077424 | 7.30E-76 | rs142565486 | T | C | 0.016753 |
| 8 | 19714837 | 0.0239806 | 0.00329184 | 3.20E-13 | rs77312736 | A | G | 0.106044 |
| 8 | 19776981 | -0.0869921 | 0.00286436 | 1.00E-200 | rs4466415 | C | A | 0.140118 |
| 8 | 19777695 | -0.168548 | 0.00650586 | 5.50E-148 | rs75218485 | T | C | 0.023587 |
| 8 | 19826373 | -0.0637172 | 0.00198763 | 1.00E-200 | rs2197089 | A | G | 0.549443 |
| 8 | 19835050 | -0.0465992 | 0.0025421 | 4.70E-75 | rs2165557 | T | A | 0.186042 |
| 8 | 19838353 | -0.065242 | 0.00667819 | 1.50E-22 | rs73600043 | T | G | 0.0225 |
| 8 | 19843748 | 0.04264 | 0.00658846 | 9.70E-11 | rs147011441 | A | G | 0.024998 |
| 8 | 19847645 | 0.054454 | 0.00925949 | 4.10E-09 | rs117956669 | G | T | 0.011507 |
| 8 | 19913833 | 0.0477592 | 0.00654152 | 2.90E-13 | rs118045108 | T | C | 0.023348 |
| 8 | 19738408 | -0.0875018 | 0.00891533 | 9.70E-23 | rs148383135 | A | G | 0.013042 |
| 8 | 19755175 | -0.0787215 | 0.00254322 | 1.00E-200 | rs73597688 | A | C | 0.18651 |
| 8 | 19757036 | -0.0772277 | 0.00874754 | 1.10E-18 | rs75240547 | C | G | 0.013054 |
| 8 | 19778142 | 0.0285317 | 0.0034706 | 2.00E-16 | rs34761945 | T | C | 0.093687 |
| 8 | 19805708 | 0.166326 | 0.00758716 | 1.60E-106 | rs1801177 | A | G | 0.017255 |
| 8 | 19823192 | 0.154168 | 0.00606855 | 2.30E-142 | rs3289 | C | T | 0.027497 |
| 8 | 19890654 | 0.0523227 | 0.00291856 | 7.20E-72 | rs4557718 | C | T | 0.132042 |
| 8 | 19710468 | 0.0316643 | 0.00201037 | 6.80E-56 | rs4244456 | C | T | 0.548311 |
| 8 | 19717091 | 0.0467802 | 0.0024477 | 2.00E-81 | rs6586874 | G | A | 0.785063 |
| 8 | 19731858 | -0.0651127 | 0.00873024 | 8.80E-14 | rs140801028 | G | C | 0.013779 |
| 8 | 19751560 | 0.0287463 | 0.00198414 | 1.40E-47 | rs1441776 | G | C | 0.510501 |
| 8 | 19756813 | -0.0391045 | 0.00203781 | 4.50E-82 | rs10102717 | T | C | 0.392711 |
| 8 | 19813529 | 0.226897 | 0.00739462 | 1.00E-200 | rs268 | G | A | 0.018189 |
| 8 | 19817476 | -0.159413 | 0.00698442 | 2.60E-115 | rs308 | G | T | 0.020597 |
| 8 | 19822741 | -0.156954 | 0.00576245 | 2.30E-163 | rs117910839 | A | T | 0.031406 |
| 8 | 19830921 | -0.171506 | 0.00299412 | 1.00E-200 | rs10096633 | T | C | 0.124384 |
| 8 | 19837269 | -0.0410132 | 0.00451414 | 1.00E-19 | rs117303935 | T | C | 0.052188 |
| 8 | 19854773 | -0.0470839 | 0.00273013 | 1.20E-66 | rs2410622 | C | T | 0.839014 |
| 8 | 19891915 | -0.071252 | 0.00931953 | 2.10E-14 | rs144014029 | C | T | 0.011457 |
| 8 | 19710388 | 0.0178256 | 0.00276695 | 1.20E-10 | rs4922108 | C | T | 0.15026 |
| 8 | 19727569 | 0.0346894 | 0.00463312 | 7.00E-14 | rs17091574 | C | T | 0.049222 |
| 8 | 19774005 | 0.0377649 | 0.00666587 | 1.50E-08 | rs148048657 | A | G | 0.025278 |
| 8 | 19825055 | -0.0399801 | 0.00544845 | 2.20E-13 | rs113831503 | T | C | 0.034267 |
| 8 | 19871320 | -0.0651913 | 0.0019904 | 1.00E-200 | rs1372343 | T | C | 0.452231 |
| 8 | 19888313 | 0.0472318 | 0.00367803 | 9.60E-38 | rs73208821 | C | G | 0.078857 |
| 8 | 19910576 | -0.0531426 | 0.00603306 | 1.30E-18 | rs148754782 | A | G | 0.027908 |
| 8 | 19912060 | -0.0807274 | 0.00219557 | 1.00E-200 | rs66462329 | A | G | 0.283325 |

**Supplementary Table 10.** The detail of instrumental variable corresponding to APOC3.

| chr.exposure | pos.exposure | beta.exposure | se.exposure | pval.exposure | SNP | effect_allele.exposure | other_allele.exposure | eaf.exposure |
| --- | --- | --- | --- | --- | --- | --- | --- | --- |
| 11 | 116610294 | -0.0729835 | 0.00257629 | 1.50E-176 | rs61905084 | C | T | 0.180713 |
| 11 | 116623659 | -0.0937927 | 0.00207286 | 1.00E-200 | rs180327 | T | C | 0.645279 |
| 11 | 116629555 | -0.0566905 | 0.00652029 | 3.50E-18 | rs150233369 | A | G | 0.02459 |
| 11 | 116675294 | -0.112042 | 0.00303292 | 1.00E-200 | rs6589570 | T | A | 0.875877 |
| 11 | 116677048 | -0.0462866 | 0.00681198 | 1.10E-11 | rs12802202 | C | T | 0.02272 |
| 11 | 116700169 | -0.0251343 | 0.002071 | 6.80E-34 | rs2854116 | T | C | 0.645289 |
| 11 | 116710968 | -0.0793887 | 0.00220781 | 1.00E-200 | rs613808 | G | A | 0.715249 |
| 11 | 116774447 | -0.0387731 | 0.00379278 | 1.60E-24 | rs34144542 | G | A | 0.075127 |
| 11 | 116606766 | -0.245288 | 0.00393661 | 1.00E-200 | rs1974718 | A | G | 0.931914 |
| 11 | 116609540 | -0.0425397 | 0.00448663 | 2.50E-21 | rs11216122 | T | G | 0.05143 |
| 11 | 116639692 | -0.0688862 | 0.00209055 | 1.00E-200 | rs1268353 | T | C | 0.342494 |
| 11 | 116645275 | -0.131603 | 0.0023033 | 1.00E-200 | rs7118999 | T | C | 0.753003 |
| 11 | 116645336 | -0.0751594 | 0.00753946 | 2.10E-23 | rs3087611 | T | A | 0.017798 |
| 11 | 116649131 | -0.050457 | 0.00690954 | 2.80E-13 | rs111732554 | C | G | 0.02099 |
| 11 | 116650571 | -0.0615155 | 0.00828196 | 1.10E-13 | rs113271699 | A | C | 0.014811 |
| 11 | 116669428 | -0.0420515 | 0.00260213 | 9.60E-59 | rs35412484 | C | T | 0.176984 |
| 11 | 116679155 | -0.0615272 | 0.00393819 | 5.10E-55 | rs75542613 | A | G | 0.068647 |
| 11 | 116699395 | -0.0703707 | 0.00566642 | 2.10E-35 | rs12721078 | A | C | 0.031903 |
| 11 | 116702778 | 0.0603138 | 0.00810637 | 1.00E-13 | rs5132 | T | C | 0.015423 |
| 11 | 116752219 | -0.119998 | 0.00332452 | 1.00E-200 | rs7124741 | T | A | 0.900821 |
| 11 | 116797442 | -0.0401412 | 0.00482736 | 9.10E-17 | rs112701434 | C | T | 0.045239 |
| 11 | 116628057 | 0.209513 | 0.00392047 | 1.00E-200 | rs61905112 | A | G | 0.068441 |
| 11 | 116682668 | 0.131801 | 0.00826334 | 2.80E-57 | rs149137426 | A | T | 0.015036 |
| 11 | 116699389 | -0.213096 | 0.00537509 | 1.00E-200 | rs10790164 | G | A | 0.966364 |
| 11 | 116730833 | -0.045801 | 0.0023951 | 1.60E-81 | rs545274 | T | C | 0.220687 |
| 11 | 116749084 | -0.0404332 | 0.00727679 | 2.80E-08 | rs146092979 | A | C | 0.019597 |
| 11 | 116759332 | 0.0665803 | 0.00316654 | 3.80E-98 | rs1473177 | C | T | 0.110588 |
| 11 | 116649759 | -0.0498568 | 0.00720256 | 4.50E-12 | rs74773964 | C | T | 0.019233 |
| 11 | 116661488 | 0.110289 | 0.00638576 | 7.80E-67 | rs3135507 | T | C | 0.024962 |
| 11 | 116674818 | -0.0512718 | 0.00211714 | 1.50E-129 | rs1729408 | G | A | 0.34001 |
| 11 | 116676590 | -0.0459859 | 0.00735965 | 4.10E-10 | rs4080140 | A | G | 0.018845 |
| 11 | 116635951 | -0.0342105 | 0.00529141 | 1.00E-10 | rs79610135 | C | T | 0.03647 |
| 11 | 116667545 | -0.0970207 | 0.00456283 | 2.50E-100 | rs75919952 | T | C | 0.050151 |
| 11 | 116693871 | 0.185471 | 0.00788062 | 1.80E-122 | rs12721041 | T | C | 0.016054 |
| 11 | 116748314 | -0.0338415 | 0.00385233 | 1.60E-18 | rs681524 | C | T | 0.072077 |
| 11 | 116771356 | -0.11614 | 0.00678782 | 1.30E-65 | rs595137 | T | C | 0.977799 |

**Supplementary Table 11.** The detail of instrumental variable corresponding to ANGPTL3.

| chr.exposure | pos.exposure | beta.exposure | se.exposure | pval.exposure | SNP | effect_allele.exposure | other_allele.exposure | eaf.exposure |
| --- | --- | --- | --- | --- | --- | --- | --- | --- |
| 1 | 63028087 | -0.0556109 | 0.00607088 | 5.20E-20 | rs148662400 | C | T | 0.027936 |
| 1 | 63092886 | 0.042028 | 0.00502409 | 6.00E-17 | rs79152165 | A | G | 0.043406 |
| 1 | 63105389 | 0.0246224 | 0.00375021 | 5.20E-11 | rs61775945 | T | C | 0.078401 |
| 1 | 63121468 | -0.0640714 | 0.00802046 | 1.40E-15 | rs112548469 | C | T | 0.017329 |
| 1 | 63158722 | 0.0335124 | 0.0022468 | 2.60E-50 | rs34693359 | C | T | 0.267904 |
| 1 | 62991248 | -0.0535773 | 0.00687062 | 6.30E-15 | rs138783696 | C | T | 0.022275 |
| 1 | 63010311 | -0.0669559 | 0.00777305 | 7.10E-18 | rs61775912 | G | A | 0.017879 |
| 1 | 63091471 | -0.055166 | 0.00292798 | 3.50E-79 | rs11207998 | C | G | 0.13239 |
| 1 | 63095677 | 0.0481747 | 0.00777719 | 5.90E-10 | rs78942743 | T | C | 0.016888 |
| 1 | 63103217 | -0.0773763 | 0.00207734 | 1.00E-200 | rs1570694 | G | A | 0.350981 |
| 1 | 62974656 | 0.0289817 | 0.00328084 | 1.00E-18 | rs61775882 | C | T | 0.107758 |
| 1 | 63028198 | 0.0369329 | 0.0022715 | 1.90E-59 | rs12749263 | C | T | 0.262393 |
| 1 | 63060802 | -0.0528548 | 0.00653548 | 6.10E-16 | rs79151558 | G | A | 0.023822 |
| 1 | 63105425 | -0.0495702 | 0.00451652 | 5.00E-28 | rs76272805 | A | G | 0.050876 |
| 1 | 63130601 | -0.0499004 | 0.00496823 | 9.80E-24 | rs74716393 | C | T | 0.042351 |
| 1 | 63002551 | 0.0774978 | 0.00207528 | 1.00E-200 | rs1168017 | G | A | 0.647608 |
| 1 | 63129802 | 0.0366026 | 0.00506378 | 4.90E-13 | rs115436978 | A | G | 0.040313 |
| 1 | 63132397 | -0.0548745 | 0.00597021 | 3.90E-20 | rs17388017 | T | A | 0.0289 |
| 1 | 63008190 | -0.0666018 | 0.00849453 | 4.50E-15 | rs116423924 | T | G | 0.014449 |
| 1 | 63038147 | 0.0536714 | 0.00732586 | 2.40E-13 | rs564992513 | C | G | 0.031435 |
| 1 | 63140536 | 0.0290383 | 0.00445198 | 6.90E-11 | rs12737254 | G | A | 0.05262 |

**Supplementary Table 12.** The detail of instrumental variable corresponding to PPARA.

| chr.exposure | pos.exposure | beta.exposure | se.exposure | pval.exposure | SNP | effect_allele.exposure | other_allele.exposure | eaf.exposure |
| --- | --- | --- | --- | --- | --- | --- | --- | --- |
| 22 | 46682310 | 0.0233454 | 0.00327525 | 1.00E-12 | rs9615934 | G | C | 0.103337 |
| 22 | 46619419 | 0.0178326 | 0.00243641 | 2.50E-13 | rs6008191 | G | A | 0.218723 |

**Supplementary Table 13.** The result of heterogeneity test and horizontal pleiotropic test.

| Outcomes | Drug Target | Heterogeneity test | | | | Horizontal pleiotropic test | | |
| --- | --- | --- | --- | --- | --- | --- | --- | --- |
|  |  | Method | Q | Q_df | Q_pval | egger_intercept | SE | *p*-value |
| CKD (CKDGen database) | LDLR | MR Egger | 38.95651028 | 38 | 0.426528208 | 0.004834995 | 0.004582155 | 0.298006246 |
|  |  | Inverse variance weighted | 40.09793915 | 39 | 0.421299247 |  |  |  |
|  | HMGCR | MR Egger | 8.226204901 | 13 | 0.82855743 | 0.006517262 | 0.01383338 | 0.645358862 |
|  |  | Inverse variance weighted | 8.448164444 | 14 | 0.864696724 |  |  |  |
|  | PCSK9 | MR Egger | 19.22552871 | 25 | 0.786180479 | 0.007551266 | 0.004924769 | 0.137754824 |
|  |  | Inverse variance weighted | 21.57661122 | 26 | 0.71162379 |  |  |  |
|  | NPC1L1 | MR Egger | 7.141434201 | 4 | 0.12860019 | 0.014180656 | 0.020908517 | 0.534837935 |
|  |  | Inverse variance weighted | 7.962677088 | 5 | 0.158304597 |  |  |  |
|  | APOB | MR Egger | 26.55041476 | 25 | 0.378710968 | 0.002249197 | 0.007440624 | 0.76493463 |
|  |  | Inverse variance weighted | 26.64745853 | 26 | 0.427996248 |  |  |  |
|  | ABCG5 | MR Egger | 12.28205337 | 14 | 0.583662997 | 0.003957639 | 0.007685875 | 0.614639723 |
|  |  | Inverse variance weighted | 12.54719971 | 15 | 0.637226923 |  |  |  |
|  | ABCG8 | MR Egger | 10.63924007 | 13 | 0.641010796 | 0.004549802 | 0.007699749 | 0.564718356 |
|  |  | Inverse variance weighted | 10.98840605 | 14 | 0.686946544 |  |  |  |
|  | LPL | MR Egger | 34.95751714 | 41 | 0.735180277 | 0.006158353 | 0.004171832 | 0.147536631 |
|  |  | Inverse variance weighted | 37.13660933 | 42 | 0.684059729 |  |  |  |
|  | APOC3 | MR Egger | 23.85967216 | 29 | 0.735820139 | -0.007793657 | 0.00543766 | 0.162473394 |
|  |  | Inverse variance weighted | 25.9139469 | 30 | 0.679516609 |  |  |  |
|  | ANGPTL3 | MR Egger | 11.73096213 | 16 | 0.762283059 | 0.016689287 | 0.011633715 | 0.1706691 |
|  |  | Inverse variance weighted | 13.78893062 | 17 | 0.681982357 |  |  |  |
| CKD (FinnGen database) | LDLR | MR Egger | 59.2884496 | 41 | 0.03211372 | 0.010176179 | 0.012549448 | 0.422113094 |
|  |  | Inverse variance weighted | 60.23928748 | 42 | 0.033711715 |  |  |  |
|  | HMGCR | MR Egger | 11.98684157 | 17 | 0.800933359 | 0.001135414 | 0.028813236 | 0.969025781 |
|  |  | Inverse variance weighted | 11.98839441 | 18 | 0.847836098 |  |  |  |
|  | PCSK9 | MR Egger | 19.5949136 | 27 | 0.847318391 | -0.012638219 | 0.009789112 | 0.207631889 |
|  |  | Inverse variance weighted | 21.26172015 | 28 | 0.814269228 |  |  |  |
|  | NPC1L1 | MR Egger | 3.143098256 | 4 | 0.534170268 | 0.021571126 | 0.037626436 | 0.597134096 |
|  |  | Inverse variance weighted | 3.471767819 | 5 | 0.627663757 |  |  |  |
|  | APOB | MR Egger | 11.96606205 | 28 | 0.99645886 | -0.00793437 | 0.017913221 | 0.661216932 |
|  |  | Inverse variance weighted | 12.16225234 | 29 | 0.997435419 |  |  |  |
|  | ABCG5 | MR Egger | 25.74658058 | 17 | 0.079202984 | -0.033662413 | 0.021616294 | 0.137826515 |
|  |  | Inverse variance weighted | 29.41939201 | 18 | 0.043488331 |  |  |  |
|  | ABCG8 | MR Egger | 24.57404752 | 16 | 0.077694934 | -0.032796103 | 0.021790857 | 0.151798476 |
|  |  | Inverse variance weighted | 28.05303267 | 17 | 0.044322349 |  |  |  |
|  | LPL | MR Egger | 38.89270185 | 46 | 0.761921083 | 0.023176835 | 0.010421452 | 0.031101133 |
|  |  | Inverse variance weighted | 43.83867505 | 47 | 0.604285398 |  |  |  |
|  | APOC3 | MR Egger | 42.82327344 | 33 | 0.117650875 | -0.002162052 | 0.015729062 | 0.89150572 |
|  |  | Inverse variance weighted | 42.84779187 | 34 | 0.142037042 |  |  |  |
|  | ANGPTL3 | MR Egger | 12.42237139 | 18 | 0.824682333 | 0.032810766 | 0.029025088 | 0.27313432 |
|  |  | Inverse variance weighted | 13.70023841 | 19 | 0.80087699 |  |  |  |
|  | PPARA | Inverse variance weighted | 0.159492307 | 1 | 0.689624368 |  |  |  |
| CKD (ebi database) | LDLR | MR Egger | 2.968020258 | 8 | 0.936348373 | -0.003864263 | 0.009127944 | 0.68319567 |
|  |  | Inverse variance weighted | 3.147240641 | 9 | 0.958169907 |  |  |  |
|  | HMGCR | MR Egger | 1.754321799 | 6 | 0.940850964 | 0.019848356 | 0.028183641 | 0.507675153 |
|  |  | Inverse variance weighted | 2.250291182 | 7 | 0.944699251 |  |  |  |
|  | PCSK9 | MR Egger | 10.26981106 | 5 | 0.067941422 | 0.006824823 | 0.038852282 | 0.867452958 |
|  |  | Inverse variance weighted | 10.33318959 | 6 | 0.111303893 |  |  |  |
|  | NPC1L1 | MR Egger | 3.018693007 | 3 | 0.388752052 | -0.02943651 | 0.028222933 | 0.373601437 |
|  |  | Inverse variance weighted | 4.113319747 | 4 | 0.390886869 |  |  |  |
|  | APOB | MR Egger | 22.70978136 | 11 | 0.019416376 | 0.012698968 | 0.019844717 | 0.535327541 |
|  |  | Inverse variance weighted | 23.55519119 | 12 | 0.023365723 |  |  |  |
|  | ABCG5 | MR Egger | 7.35771836 | 7 | 0.392607974 | -0.019888625 | 0.015000067 | 0.226499963 |
|  |  | Inverse variance weighted | 9.205574927 | 8 | 0.325251958 |  |  |  |
|  | ABCG8 | MR Egger | 7.35771836 | 7 | 0.392607974 | -0.019888625 | 0.015000067 | 0.226499963 |
|  |  | Inverse variance weighted | 9.205574927 | 8 | 0.325251958 |  |  |  |
|  | LPL | MR Egger | 13.37409633 | 17 | 0.710804787 | 0.000997435 | 0.008145525 | 0.903977112 |
|  |  | Inverse variance weighted | 13.38909078 | 18 | 0.767959451 |  |  |  |
|  | APOC3 | MR Egger | 0.895610256 | 5 | 0.970538628 | -0.01143529 | 0.019311216 | 0.579505908 |
|  |  | Inverse variance weighted | 1.246261284 | 6 | 0.974538096 |  |  |  |
|  | ANGPTL3 | MR Egger | 4.556117399 | 2 | 0.102482964 | 0.033898458 | 0.049243063 | 0.562331346 |
|  |  | Inverse variance weighted | 5.635647352 | 3 | 0.130746796 |  |  |  |

**Supplementary Table 14.** The result of heterogeneity test and horizontal pleiotropic test.

| Outcomes | Drug Target | Heterogeneity test | | | | Horizontal pleiotropic test | | |
| --- | --- | --- | --- | --- | --- | --- | --- | --- |
|  |  | Method | Q | Q_df | Q_pval | egger_intercept | SE | *p*-value |
| eGFR (CKDGen database) | LDLR | MR Egger | 53.48965645 | 38 | 0.049006056 | 3.59E-05 | 0.000198049 | 0.85705037 |
|  |  | Inverse variance weighted | 53.53595425 | 39 | 0.060532237 |  |  |  |
|  | HMGCR | MR Egger | 4.376200866 | 13 | 0.98649186 | -0.000498349 | 0.000509401 | 0.345779371 |
|  |  | Inverse variance weighted | 5.333279946 | 14 | 0.980612475 |  |  |  |
|  | PCSK9 | MR Egger | 49.40725496 | 25 | 0.002519889 | -0.000422715 | 0.000249332 | 0.102423447 |
|  |  | Inverse variance weighted | 55.08777587 | 26 | 0.000738942 |  |  |  |
|  | NPC1L1 | MR Egger | 7.270066677 | 4 | 0.122286339 | 0.000341132 | 0.000792556 | 0.689067618 |
|  |  | Inverse variance weighted | 7.606781201 | 5 | 0.179279707 |  |  |  |
|  | APOB | MR Egger | 18.4318727 | 25 | 0.823517312 | -0.000447275 | 0.00026829 | 0.10796901 |
|  |  | Inverse variance weighted | 21.21120115 | 26 | 0.730981437 |  |  |  |
|  | ABCG5 | MR Egger | 7.733400386 | 14 | 0.902747627 | -0.000458305 | 0.000285113 | 0.130267904 |
|  |  | Inverse variance weighted | 10.31728993 | 15 | 0.799321117 |  |  |  |
|  | ABCG8 | MR Egger | 7.733386754 | 13 | 0.860525345 | -0.00045825 | 0.000285498 | 0.132481549 |
|  |  | Inverse variance weighted | 10.30969867 | 14 | 0.739208564 |  |  |  |
|  | LPL | MR Egger | 22.50466682 | 41 | 0.99163456 | -0.000404754 | 0.000157695 | 0.014016121 |
|  |  | Inverse variance weighted | 29.09252675 | 42 | 0.934582676 |  |  |  |
|  | APOC3 | MR Egger | 20.52463917 | 29 | 0.87587298 | -0.000241468 | 0.000204046 | 0.246259803 |
|  |  | Inverse variance weighted | 21.9250827 | 30 | 0.856755287 |  |  |  |
|  | ANGPTL3 | MR Egger | 9.476112325 | 16 | 0.892519568 | -0.000384887 | 0.000428755 | 0.382662926 |
|  |  | Inverse variance weighted | 10.28195127 | 17 | 0.891372135 |  |  |  |
| BUN (CKDGen database) | LDLR | MR Egger | 28.70799727 | 37 | 0.833471081 | -4.93E-05 | 0.0004422 | 0.911901008 |
|  |  | Inverse variance weighted | 28.72040731 | 38 | 0.861650786 |  |  |  |
|  | HMGCR | MR Egger | 13.48355253 | 13 | 0.411191873 | 0.000124307 | 0.001367333 | 0.92894832 |
|  |  | Inverse variance weighted | 13.49212497 | 14 | 0.48819108 |  |  |  |
|  | PCSK9 | MR Egger | 54.02574175 | 24 | 0.000422887 | 0.00102886 | 0.000777659 | 0.198295351 |
|  |  | Inverse variance weighted | 57.96599684 | 25 | 0.000198162 |  |  |  |
|  | NPC1L1 | MR Egger | 1.623668661 | 4 | 0.804531985 | -0.000372445 | 0.001462765 | 0.811573205 |
|  |  | Inverse variance weighted | 1.688498538 | 5 | 0.89034555 |  |  |  |
|  | APOB | MR Egger | 14.25235119 | 23 | 0.919416369 | -0.000837455 | 0.000691926 | 0.238446258 |
|  |  | Inverse variance weighted | 15.7172376 | 24 | 0.898010833 |  |  |  |
|  | ABCG5 | MR Egger | 14.47044619 | 14 | 0.41528242 | 0.000704243 | 0.000734243 | 0.353759986 |
|  |  | Inverse variance weighted | 15.42131214 | 15 | 0.421516428 |  |  |  |
|  | ABCG8 | MR Egger | 13.61740365 | 13 | 0.401329029 | 0.000668654 | 0.000740211 | 0.382785358 |
|  |  | Inverse variance weighted | 14.47215995 | 14 | 0.415159379 |  |  |  |
|  | LPL | MR Egger | 35.90955034 | 41 | 0.695941408 | -0.000110435 | 0.000397288 | 0.782431283 |
|  |  | Inverse variance weighted | 35.98681847 | 42 | 0.731245933 |  |  |  |
|  | APOC3 | MR Egger | 28.4527064 | 29 | 0.493818534 | 0.000601641 | 0.000531273 | 0.266721345 |
|  |  | Inverse variance weighted | 29.73515366 | 30 | 0.479276151 |  |  |  |
|  | ANGPTL3 | MR Egger | 12.71586302 | 16 | 0.693408333 | 0.000681173 | 0.001133709 | 0.55636575 |
|  |  | Inverse variance weighted | 13.07686704 | 17 | 0.731025227 |  |  |  |

**Supplementary Table 15.** The result of heterogeneity test and horizontal pleiotropic test (adjusted).

| Outcomes | Drug Target | Heterogeneity test | | | | Horizontal pleiotropic test | | |
| --- | --- | --- | --- | --- | --- | --- | --- | --- |
|  |  | Method | Q | Q_df | Q_pval | egger_intercept | SE | p-value |
| CKD (FinnGen database) | LPL | MR Egger | 24.30820291 | 35 | 0.912394291 | 0.012313457 | 0.012071031 | 0.314688593 |
|  |  | Inverse variance weighted | 25.34877281 | 36 | 0.907467252 |  |  |  |
| eGFR (FinnGen database) | LPL | MR Egger | 21.46446729 | 30 | 0.872804681 | -0.000198876 | 0.000185176 | 0.291391501 |
|  |  | Inverse variance weighted | 22.61791462 | 31 | 0.86265599 |  |  |  |

**Supplementary Table 16.** The results of Mediation Analysis.

| outcome | exposure | method | nsnp | b | se | pval | orDrug | lci95Drug | uci95Drug |
| --- | --- | --- | --- | --- | --- | --- | --- | --- | --- |
| HLA DR on CD14+ CD16+ monocyte \|\| id:ebi-a-GCST90002007 | ANGPTL3 | MR Egger | 20 | -0.192450344 | 0.554864541 | 0.732731907 | 1.212216309 | 0.408573213 | 3.596585217 |
| HLA DR on CD14+ CD16+ monocyte \|\| id:ebi-a-GCST90002007 | ANGPTL3 | Weighted median | 20 | -0.458834693 | 0.262991823 | 0.081041832 | 1.582229129 | 0.944944315 | 2.649308512 |
| HLA DR on CD14+ CD16+ monocyte \|\| id:ebi-a-GCST90002007 | ANGPTL3 | Inverse variance weighted | 20 | -0.458996088 | 0.213962761 | 0.031935674 | 1.582484512 | 1.040424784 | 2.406956532 |
| HLA DR on CD14+ CD16+ monocyte \|\| id:ebi-a-GCST90002007 | ANGPTL3 | Simple mode | 20 | -0.484189709 | 0.39982527 | 0.240742561 | 1.622859487 | 0.741212574 | 3.553195139 |
| HLA DR on CD14+ CD16+ monocyte \|\| id:ebi-a-GCST90002007 | ANGPTL3 | Weighted mode | 20 | -0.484189709 | 0.270972589 | 0.089926446 | 1.622859487 | 0.954167005 | 2.760180243 |
|  |  |  |  |  |  |  |  |  |  |
| exposure | outcome | method | nsnp | b | se | pval | or | or_lci95 | or_uci95 |
| HLA DR on CD14+ CD16+ monocyte | Chronic kidney disease | MR Egger | 16 | -0.011948106 | 0.008777861 | 0.194969692 | 0.988122989 | 0.971268127 | 1.005270341 |
| HLA DR on CD14+ CD16+ monocyte | Chronic kidney disease | Weighted median | 16 | -0.016447364 | 0.009713863 | 0.090420307 | 0.983687156 | 0.96513573 | 1.002595168 |
| HLA DR on CD14+ CD16+ monocyte | Chronic kidney disease | Inverse variance weighted | 16 | -0.019584773 | 0.007002804 | 0.005162677 | 0.980605763 | 0.967238408 | 0.994157856 |
| HLA DR on CD14+ CD16+ monocyte | Chronic kidney disease | Simple mode | 16 | -0.040088656 | 0.021116261 | 0.07704471 | 0.960704263 | 0.921754346 | 1.001300059 |
| HLA DR on CD14+ CD16+ monocyte | Chronic kidney disease | Weighted mode | 16 | -0.018113904 | 0.008781544 | 0.056898506 | 0.982049166 | 0.965290941 | 0.999098328 |
|  |  |  |  |  |  |  |  |  |  |
| exposure | outcome | method | nsnp | b | se | pval | orDrug | lci95Drug | uci95Drug |
| ANGPTL3 | Chronic kidney disease | MR Egger | 18 | -0.082009982 | 0.204664874 | 0.693938183 | 1.085466645 | 0.726778757 | 1.621178144 |
| ANGPTL3 | Chronic kidney disease | Weighted median | 18 | 0.155518037 | 0.089211195 | 0.08128874 | 0.855971637 | 0.718656358 | 1.019524055 |
| ANGPTL3 | Chronic kidney disease | Inverse variance weighted | 18 | -0.191692011 | 0.074070137 | 0.009654067 | 0.825561093 | 0.714002026 | 0.954550678 |
| ANGPTL3 | Chronic kidney disease | Simple mode | 18 | 0.289212844 | 0.161886102 | 0.091860904 | 0.748852799 | 0.545250692 | 1.028481987 |
| ANGPTL3 | Chronic kidney disease | Weighted mode | 18 | 0.171719012 | 0.08285502 | 0.053736686 | 0.842215792 | 0.715971548 | 0.990720153 |
